# Pressure–Volume Curve during Capnoperitoneum in Cats

**DOI:** 10.3390/ani10081408

**Published:** 2020-08-13

**Authors:** Melissa Dorn, Anja Becher-Deichsel, Barbara Bockstahler, Christian Peham, Gilles Dupré

**Affiliations:** 1 Clinic for Small Animals, Division of Small Animal Surgery, University of Veterinary Medicine Vienna, 1210 Vienna, Austria; barbara.bockstahler@vetmeduni.ac.at (B.B.); gilles.dupre@vetmeduni.ac.at (G.D.); 2 Clinic for Obstetrics, Division of Gynecology and Andrology, University of Veterinary Medicine Vienna, 1210 Vienna, Austria; anjabech@web.de; 3 Clinic for Horses, Division of Movement Science Group, University of Veterinary Medicine, 1210 Vienna, Austria; christian.peham@vetmeduni.ac.at

**Keywords:** cats, feline, pressure–volume curve, capnoperitoneum, intra-abdominal pressure, laparoscopy, minimal invasive surgery, keyhole surgery, endoscopic surgery, veterinary laparoscopic guidelines

## Abstract

**Simple Summary:**

Keyhole surgery has become a very popular form of surgery in human and veterinary medicine because of its advantages in patient recovery and smaller incision sites. The disadvantages are side effects of high intra-abdominal pressures on the circulation system. Veterinary surgeons initially adapted guidelines from human medicine with pressure limits of 15 mmHg, although little is known about pressure limits in smaller sized patients and differences in species. Most surgeons already use smaller pressure limits but very few studies exist and guidelines are often based on personal experience. This study analyzes the pressure relationship in the abdomen during keyhole surgery for neutering in 59 cats by measuring the volume inflated and the pressure from 0 to 15 mmHg and compares it to previous study results in dogs. This study shows that, similar to dogs, the intra-abdominal pressure and the insufflated volume rise until a threshold of 6.44 mmHg is reached. After this point, the volume gain decreases and pressure rises exponentially with each additional insufflation. This means insufflation over 7 mmHg in cats will result in minimal gain and should be avoided.

**Abstract:**

Laparoscopy is a growing field in veterinary medicine, although guidelines are lacking. The objective of this study was to evaluate the pressure–volume curve during capnoperitoneum in cats. A total of 59 female cats were scheduled for routine laparoscopy. Pressure and volume data were recorded and processed, and the yield point of the curve was calculated using a method based on a capacitor discharging function. For the remaining 40 cats, a linear-like pressure–volume curve was observed until a yield point with a mean cutoff pressure (COP) of 6.44 ± 1.7 mmHg (SD) (range, 2.72–13.00 mmHg) and a mean cutoff volume (COV) of 387 ± 144.35 mL (SD) (range, 178.84–968.43 mL) was reached. The mean mL/kg CO_2_ value in cats was 208 ± 34.69 mL/kg (range, 100.00–288.46 mL/kg). The COV correlated with COP and body weight but not with body condition score (BCS). COP correlated only with the COV. This study suggests that feline patients have a pressure–volume curve similar to that of canine patients, and the same pressure limit recommendations can be used for both species. After a yield point of 6.44 mmHg is reached, the increment in volume decreases exponentially as the intra-abdominal pressure (IAP) increases.

## 1. Introduction

The use of laparoscopy in small animal surgery has greatly increased over the last 15 years and has been employed in canine as well as in feline patients [1,2,3,4,5,6,7,8,9,10,11,12,13]. The advantages of this technique include reduced postoperative pain, shorter postoperative recovery periods, and less wound healing complications [14,15,16,17,18].

During laparoscopy, the abdomen is usually insufflated with CO_2_, creating a capnoperitoneum to achieve adequate working space for the safe performance of laparoscopic surgery. However, the created space depends mainly on the injected volume and not on the IAP predetermined by the surgeon. Current guidelines adopted from human medicine recommend pressure levels of 15 mmHg or lower to avoid complications of high intra-abdominal pressure (IAP) [19]. These complications include cardiovascular parameters such as hypotension and hypoxemia due to decreased venous return, decreased cardiac output and ventilation capacity, and metabolic changes due to reduced organ perfusion and consequent possible organ failure [20,21,22,23,24,25,26,27,28,29]. Furthermore, these guidelines do not consider the variety of sizes of veterinary patients. In order to perform safe laparoscopic procedures, the surgeon must have sufficient working space and visibility of the organs. This is especially difficult to achieve in the surgery of small veterinary patients and is very often a reason for the need for conversion to open surgery. Therefore, adequate working space is mandatory. Considering the high learning curve for laparoscopic surgeons, the most frequent and easiest way of increasing visibility in the working space is to increase the IAP. Alternative methods for more experienced and advanced laparoscopic surgeons include low IAP capnoperitoneum in combination with the wall lift method and the wall lift method alone [4,19,30,31,32]. Even within the recommended pressure levels for smaller animals, complications have been reported to occur [21,33]. Despite its importance for laparoscopy, the pressure–volume (PV) curve has, to our knowledge, been addressed in only a few studies. A clinical study in humans evaluated the influence of different recumbencies on the intra-abdominal volume (IAV) of obese patients. The study showed an increase in IAV in the beach chair position in comparison with other positions at a similar IAP [34]. Another study series in pigs examining IAV in a computed tomography model during pneumoperitoneum at different IAP levels discovered that the greatest volume gain was attained at 10 mmHg and that in piglets, 80% of IAV was already achieved at this pressure level [35,36]. One further study in cats analyzed cardiorespiratory function at three different IAP pressure levels (4, 8, and 15 mmHg) and its role in the working space in relation to circumference and height [21]. The study concluded that there was significant increase in height and circumference at 4 and 8 mmHg, and at 15 mmHg, there was a significant increase in circumference but not in height. Furthermore, the cardiorespiratory complications increased above 8 mmHg. Therefore, the authors recommended pressure levels below 8 mmHg for cats.

Besides the latter study, there exists only one other PV study in companion animal medicine [37]. This study examined 66 dogs, divided into three weight groups, comparing their PV curves. They discovered a yield point for all dogs independent of their weight, composed by a cutoff pressure (COP) of (mean ± SD) 5.99 ± 0.805 mmHg and a cutoff volume (COV) of 1196.2 ± 697.9 mL, after which the increase in IAP did not result in a significant gain of IAV. This suggests that increasing the IAP by over 6 mmHg results in increasingly less volume gain.

The study showed that the IAP behaves “linear-like” until a yield point with a COP of 5.99 ± 0.805 mmHg is reached. Beyond this pressure level, the PV curve increases exponentially. The yield pressure level does not correlate with body weight or other body variables.

In cats, as in dogs, the recommended pressure values vary greatly among work groups, for example, 4 mmHg [8]; 4–8 mmHg [21]; 6 mmHg [38]; 6–8 mmHg [4,19]; 10–12 mmHg [9,39] or 12–13 mmHg [40]. The advantages of lowering the working pressure, including less post-operative pain, have been shown in different studies of human medicine [41,42]. Negative cardiopulmonary effects were observed in cats when working with a low-pressure abdominal insufflation of 6 mmHg, and the effects worsened with longer duration of peritoneal insufflation [33]. 

Cats and small dogs have a smaller abdominal cavity than larger animals. This presents a predicament for laparoscopic surgeons who have to work in a very restricted working space. Although some authors report that the working space at a pressure level of 4 mmHg in cats is large enough to visualize all essential organs due to a greater abdominal wall flexibility, the working space has, to our knowledge, not been investigated in cats [8]. For this to happen, the relationship between IAP and IAV of cats during capnoperitoneum needs to be evaluated. The aim of our study was to evaluate the PV curve in a capnoperitoneum model and to find a yield point with a COV and COP in cats. We hypothesized that the IAV does not increase in linearity with intra-abdominal pressure and becomes exponential after a COP is reached. 

## 2. Materials and Methods

### 2.1. Approval and Consent

The study was approved by the institutional ethics committee in accordance with Good Scientific Practice guidelines and national legislation under the number 01/06/S97/2014. The owners provided informed consent for the inclusion of their pets in this study.

### 2.2. Animals

In total, 59 cats scheduled for routine ovariectomy at the Clinic for Obstetrics, Gynecology and Andrology, Veterinary University of Vienna from November 2014 to March 2016 were enrolled in the study. Age, weight, and breed were recorded for each patient. The body condition score (BCS) of each patient was determined using a 9-integer unit BCS system and recorded for 33 of the 59 logged animals [43]. Patients were excluded in cases of detrimental effects on the patient or abnormal abdominal content (e.g., uterine abnormalities, herniation, large intra-abdominal tumors or ascites, or gas leakage through the cannula), as these may distort the measurements of pressure and volume. 

### 2.3. Anesthesia and Analgesia

All patients were fasted for 12 h before anesthesia. For premedication, ketamine, medetomidine (2–5 mg/kg, 10–40 µg/kg), and methadone (0.2 mg/kg) was administered intravenously or intramuscularly prior to the induction of anesthesia with propofol (3–6 mg/kg). Anesthesia was maintained with isoflurane in oxygen. During surgery, crystalloid fluids were administered intravenously. Patients were ventilated with a tidal volume of 8–12 mL/kg, a maximal pulmonary pressure of 10 cm H_2_O, and a frequency of 14 breaths/min dependent on EtCO_2_ (volume-controlled ventilation). During surgery, meloxicam (0.1 mg/kg) was administered intravenously. Analgesia was continued by the owners by oral application of meloxicam (0.05 mg/kg) for three days following the day of surgery.

### 2.4. Preparation of Patients

Patients were prepared for surgery following clinical standards by manual emptying of the urinary bladder, clipping, washing, and antiseptic preparation of the operation site. 

### 2.5. Laparoscopic Procedure

The same method used in our previous canine study was also applied in this study [37]. Briefly, the animals were placed in dorsal horizontal recumbency using a modified Hasson technique with a blunt-tip balloon cannula ( Kii balloon blunt tip system, 12 × 100 mm, Applied Medical, Rancho Santa Margarita, Calif.) with a gel cushion attached to the cannula to minimize leakage. Traction sutures (Biosyn 2–0, Covidien Animal Health, Plymouth, Minn.) were used to access the abdominal cavity. After removal of the trocar, one finger from a size 6 latex glove was removed and used to cover the dorsal opening of the cannula to prevent leakage from the cannula valve. The lateral plug valve of the cannula was then connected using flexible silicon tubes and a 3-way stopcock to a pressure-measuring transducer (Edwards TruWave disposable pressure transducer serial interface RS232, company, Irvine, Calif.).

Insufflation occurred using an insufflator (Endo-Arthroflator insufflator, Karl-Storz Tuttlingen, Germany.), filling a syringe (Drenching syringe, 250 mL, No. 54217, Ukal Elevage, Eschbach, France) connected via a stopcock to the cannula and transducer. The patient was insufflated via a syringe with successive insufflations of CO_2_ until the limit of 15 mmHg was reached. The first seven cats were insufflated with a 250-mL syringe (Drenching syringe, 250 mL, No. 54217, Ukal Elevage, Eschbach, France) modified and calibrated to create a 125-mL syringe. The remaining 33 animals were insufflated with the same syringe (Drenching syringe, 250 mL, No. 54217, Ukal Elevage, Eschbach, France) modified and calibrated to create a 75-mL syringe, which allowed us to have more incremental steps and thus, more pressure levels. The additional transducer (Edwards TruWave disposable pressure transducer serial interface RS232, company, Irvine, Calif.) recorded the measured IAP and transmitted the pressure values to a computer (Lenovo ThinkPad X131e with Radeon HD graphics, 1.7 GHz, Lenovo, Morrisville, NC.) and software (Buzzer Datalogger, Version 2.00 alpha, Universiätsklinik für Anästhesie und Intensivmedizin, Anichstraße 35, 6020 Innsbruck, Austria) to record the values. During the measurement episodes, all stopcocks were closed toward the insufflator and syringe to avoid the interference of pressure readings.

Before the measurement started, a baseline IAP of 15 s was recorded. After each incremental CO_2_ insufflation, there was a 15 s pause before the subsequent increment until the pressure exceeded 15 mmHg. The cannula was, then, connected directly to the insufflator, and the pressure was allowed to escape through the cavity until a pressure of 8 mmHg was reached. The operation was then continued as scheduled.

### 2.6. Data Processing

**Figure 1:**
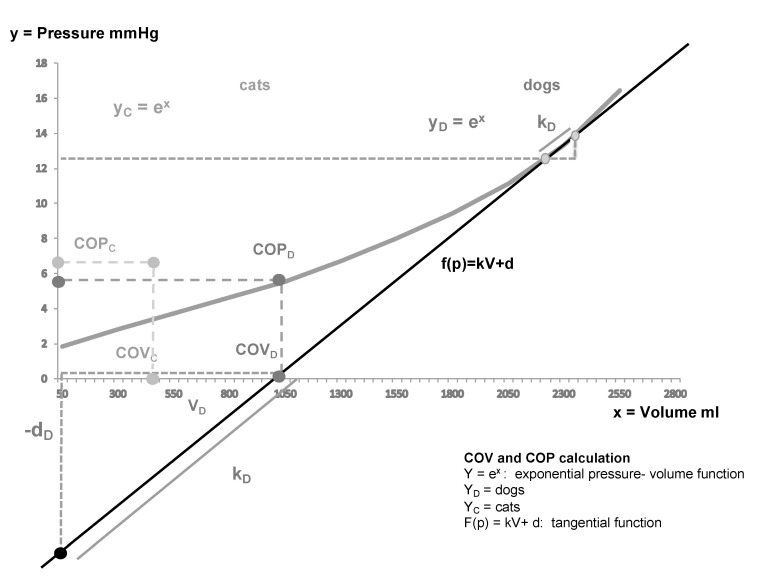
Cutoff volume (COV) and cutoff pressure (COP) calculation. Legend: The exponential PV curves (y_D_ = e^x^, y_C_ = e^x^) of dogs and cats depicting the calculation method used to define the COP and COV of the dog and cat. In order to define the COV, the two last measuring points were used to determine the slope of the tangential (black line) through these points and calculate the COV and the COP by use of the tangential function: ∫(p) = kV + d. The p represents the IAP; k is the slope of the tangent of the last two measuring points. V is the volume and is represented by the intercept of the x-axis. The intercept of the y-axis represents d. The corresponding values were inserted into the tangential formula and the intercept of the x-axis was calculated. The calculated volume represents the COV. The COP was then identified in the measured data by its corresponding COV value.

Analysis of pressure data was conducted in the same manner as described in detail in a previous dog study [37]. Briefly, the collected data were analyzed in a motion analysis program (SIMI Motion 3D, Simi Reality Motion Systems GmbH, Unterschleißheim, Germany). using 50 measuring values per increment step to calculate a mean pressure value for each step. These pressure values were plotted against their corresponding volume values, thus, forming a PV curve. This curve is represented by the exponential function y = e^x^ (Figure 1). Y represents the IAP, X is the volume of CO_2_, and e is a mathematical constant known as Euler’s number. The searched yield point of the feline PV curve was determined by the same capacitor discharging function evaluation method as in the abovementioned canine study using commercial software (Windows 7 Professional, version 6.1, Microsoft Corp, Redmond, Wash.). [37]. The slope between the last two measured points on the exponential curve was calculated and inserted into the following tangential function to identify the COP and COV points:

∫(p) = kV + d(1)

where p is the IAP, k is the slope between the last two measured points, V is the volume, and d is the intercept of the y-axis. The volume was calculated as V = –d/k; as a consequence of ∫(p) = 0, the volume was equivalent to the COV. The nearest volume value to the calculated COV was identified in the data, and its corresponding mean IAP value was chosen as the COP value, thus, providing the coordinates for the yield point on the PV curve of each cat. (Table S1: data and calculations).

### 2.7. Statistical Analyses

Statistical data were processed using commercial software data (SPSS, version 14.0, SPSS Inc., Chicago, Ill. USA). (Supplements Document 1: statistical data) To evaluate normal data distribution, Kolmogorov–Smirnov and Shapiro–Wilk tests were used. The correlations between COP and COV values and categorical parameters were evaluated using Pearson and Spearman *R*-correlation tests. Results were reported as mean ± SD, and values of *p* < 0.05 were considered significant. All the applied test assumptions were met.

## 3. Results

Of the 59 cats enrolled in the study, 19 were excluded because of gas leakage due to surgical flaws, trocar defects, or missing data due to recording or software errors. None of the animals were excluded due to surgical or anesthetic complications during the procedure. Thus, a total of 40 female cats, with a mean body weight of 2.95 ± 0.75 kg (range, 1.75–6.00 kg), were included in the study. Two of these cats were measured, but we switched to an open approach because of small body size or pathological organ formation after organ evaluation. The included cats comprised 28 European domestic short hair cats, one European domestic long hair cat, three British short hair cats, two Bengal cats, one Maine Coon cat, one Siamese cat, one Devon Rex, and one Persian cat. The mean age was 12.6 *±* 3 months (SD) (range, 4–60 months), and the mean weight was 2.95 ± 0.75 kg (SD) (range, 1.75–6.00 kg) with a mean BCS of 5.53 *±* 1.05 (SD) (range, 3–8). The PV curve in this cat study proved to have a linear-like onset and developed an exponential course after reaching a certain pressure level. The mean COP was 6.44 ± 1.7 mmHg (SD) (range, 2.72–13.00 mmHg), and the mean COV was 387 ± 144.35 mL (SD) (range, 178.84–968.43 mL) (Table 1). Except for the COV values, data were not normally distributed. Using the Pearson *R*-correlation test, we evaluated the relationship between these values and body weight and BCS. COP correlated with COV significantly (*p* < 0.01) but not with other parameters. COV correlated significantly with COP and body weight (*p* < 0.01) but not with BCS.

## 4. Discussion

In the present study, the PV curve in cats revealed a linear-like behavior until a COP of 6.44 ± 1.7 mmHg (SD) (range, 2.72–13.00 mmHg) was reached. Our previous study revealed similar findings: the PV curve in dogs behaved linear-like until a COP of 5.99 ± 0.81 mmHg (range, 4.04–8.52 mmHg) was reached [37].

However, there are relevant differences between the previous dog study and the current analysis in cats. In the current study, the COP value correlated with the COV, which was not the case in the dog study. Furthermore, the PV curve of cats reaches the yield point later than that of dogs does, acting like a linear spring (but the curve is still exponential). This finding might suggest that the abdominal wall elasticity of the cat can be represented (nearly perfectly) by a linear relation, of the pressure with respect to the volume, until the yield point.

Additionally, as depicted in Table 1, the cat group had a much higher mean CO_2_ value: 208 ± 34.69 mL/kg (range, 100.00–288.46 mL/kg) compared with the value observed in dogs in the previous study: 65.64 mL/kg CO_2_ ± 20 (range, 21.96–107.80 mL/kg) [37]. The explanation for these results could be the suspected species-specific increased tissue flexibility in cats [8]. Further studies will be necessary to properly evaluate and compare the abdominal wall compliance of cats with that of dogs. 

Another reason for these results could be related to patient size. In our previous dog study, the mean COP of the small dogs group (<11 kg; *n* = 21) was higher than the COP in the larger dogs group (>11 kg; *n* = 44) [37]. This feature could be common to smaller animals, contradicting the current guideline recommendations to limit the IAP in smaller animals in order to achieve low pressure levels of 3–4 mmHg, while allowing higher IAP levels of 13–15 mmHg in larger animals [4,8,19,21,40]. Further studies in smaller dogs or other species are necessary to evaluate this property.

In this study, CO_2_ was insufflated using a syringe method. This was also done to compare our results with our previous study in dogs [37]. However, because of the fixed volume increments of the syringe and the preset specifications in the study design to insufflate until the final pressure exceeded the limit of 15 mmHg, the pressure reached after the last syringe increment attained a mean of 19.79 ± 3.2 mmHg (range, 15.18–25.85 mmHg). These higher pressure levels at the end of the insufflation could have resulted in a wider range of COP values in the cat group than that in the dog group due to the exponential behavior of the PV curve. The syringe method used is perhaps a limitation of this study, compared with a non-incremental CO_2_ insufflation. A continuous insufflation might have been better to avoid this bias, but changing the method would also have compromised the comparability of the results to those of our first study.

As in our previous study, the use of healthy and young animals for castration could represent a limitation. How aging influences the PV curve remains unknown.

Abdominal distension has been evaluated in two human medicine studies using motion analysis markers during abdominal insufflation [44,45]. During insufflation, they observed a change from the cylindrical abdominal form to a dome-shaped form. The authors also discovered a restriction in the movement of the abdominal wall at 12 mmHg and did not continue the insufflation process beyond that value. They discovered a higher stiffness of the abdominal wall in male patients than in female patients. They additionally suspected an important role of the diaphragmatic movement in the working space because of a 25% volume difference between the software-estimated IAV and the supplied gas volume. That study did not evaluate the PV curve with regard to its ascending slope or yield point.

A limitation of the present study is that it included only female animals, which could have influenced the resulting COP value. Further studies with a more mixed population would be necessary to evaluate this further.

An earlier study in cats evaluated the working space by measuring the height and circumference of the abdomen using a tape and video images [21]. They found a significant increase in abdominal height and circumference between baseline, 4, and 8 mmHg, but only a significant increase in circumference, but not in height, between 8 and 15 mmHg. The subjective video evaluation suggested that the most evident space increase occurred between 4 and 8 mmHg, and a smaller increase occurred between 8 and 15 mmHg. The authors concluded that the working space at 4 mmHg would be enough to conclude various basic procedures, and that it is, therefore, not necessary to increase the IAP beyond 8 mmHg and run the risk of side effects (since the visible working space between 8 and 15 mmHg was similar). 

## 5. Conclusions

The results indicate that the COP for dogs and cats lies between 6 and 7 mmHg and that further insufflation results in minimal gain of volume and working space. Surgeons should avoid the use of unnecessarily high IAP and concurrent cardiovascular side effects. These results should provide the grounds for further investigations on how to increase intra-abdominal volume without increasing the IAP.

## Figures and Tables

**Table 1:** Mean ± SD values and range for Total Volume, COV, and COP of cats during intra-abdominal insufflation of CO_2_.

Variable	Mean ± SD	Range
Mean Total Volume ml	606.88 ± 167.85	375–1425
Mean COV ml	387 ± 144.35	178.84–968.43
Mean COV ml/kgCO_2_	208 ± 34.69	100–288.46
Mean COP mmHg	6.44 ± 1.7	2.72–13

COV—cutoff volume; COP—cutoff pressure.

## Supplementary Materials

The following are available online at https://www.mdpi.com/2076-2615/10/8/1408/s1, Table S1: Data and Calculations; Document 1: statistical data.
