# Supplementary material for: Pressure–Volume Curve during Capnoperitoneum in Cats
Source: Animals (Basel). 2020 Aug 13;10(8):1408. doi: 10.3390/ani10081408 (PMC7459975; doi:10.3390/ani10081408)
Supplement: Supplementary file 1 [file animals-10-01408-s001.zip › document 1 Statistical Data.pdf]

Statistics Cat Study Dorn et al English

Descriptive statistics

	N	Min	Max	Mean	SD
COP	40	2.72	13.00	6.4392	1.70082
COV	40	178.84	968.43	387.0016	144.35022
Valid values in sits	40				

Explorative data analysis: COP

Valid			Missing		Total	
COP	N	Per Cent	N	Per Cent	N	Per Cent
	40	100.0%	0	0.0%	40	100.0%

Boxplot COP

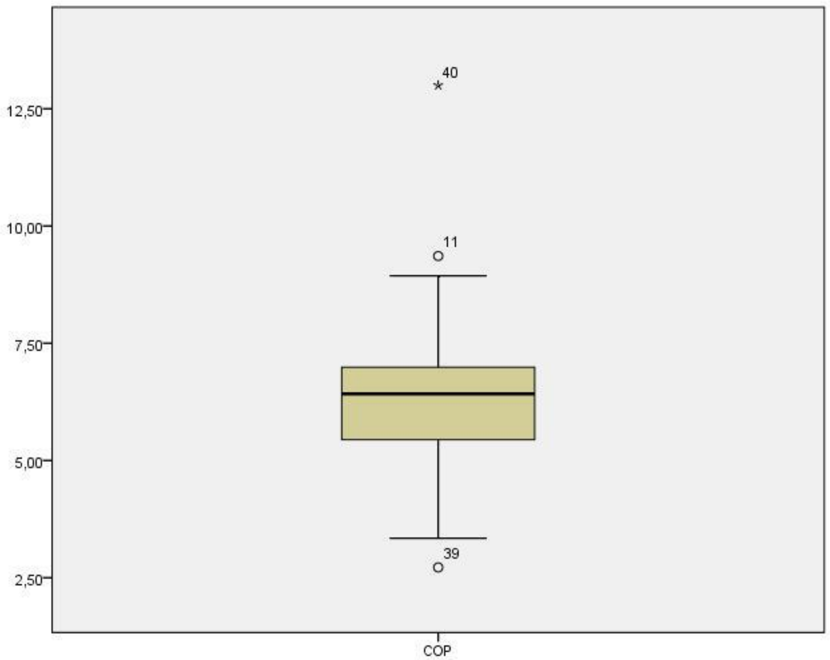

Explorative data analysis: COV

Valid			Missing		Total	
COV	N	Per Cent	N	Per Cent	N	Per Cent
	40	100.0%	0	0.0%	40	100.0%

Boxplot COV

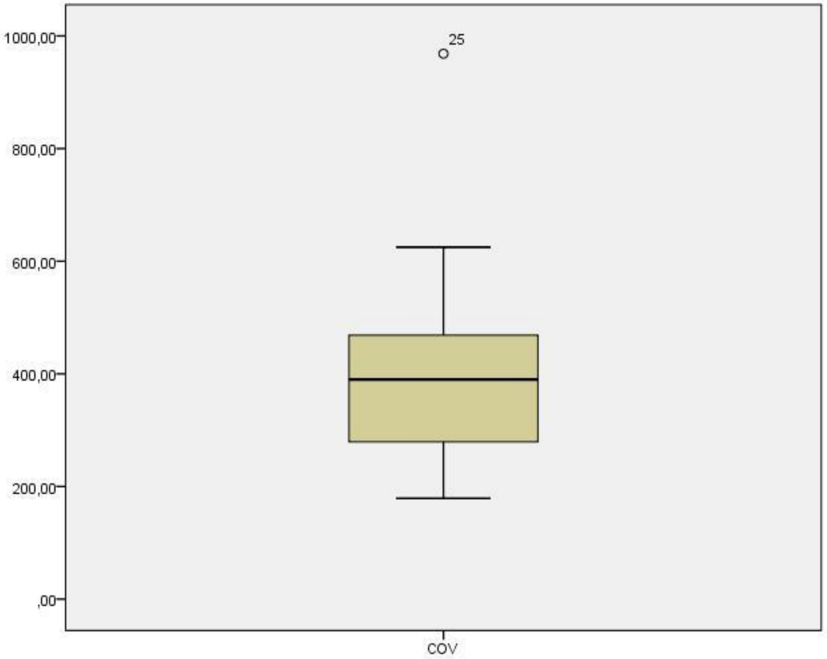

Correlations:

Descriptive statistics

	Mean	SD	N
Body weight	2.951	.7527	40
COP	6.4393	1.70082	40

Descriptive statistics COP/ COV/BW

	Mean	SD	N
COP	6.4393	1.70082	40
COV	387.0016	144.35022	40
Body weight	2.95	.7527	40

Correlations COP/COV/BW

		COP	COV	Body weight
COP	Correlation Pearson	1	.410**	.075
	Significance (two sides)		.009	.646
	N	40	40	40
COV	Correlation Pearson	.410**	1	.522**
	Significance (two sides)	.009		.001
	N	40	40	40
Body weight	Correlation Pearson	.075	.522**	1
	Significance (two sides)	.646	.001	
	N	40	40	40

** The correlation is significant (2 sides) at a level of 0.01

Spearman Correlation COP/ COV

			COP	COV
Spearman- Rho	COP	Correlation coefficient	1.000	.436**
		Sig.(2 sides)	.	.005
		N	40	40
	COV	Correlation coefficient	.436**	1.000
		Sig.(2 sides)	.005	.
		N	40	40

** The correlation is significant (2 sides) at a level of 0.01

Scatterplot COP / COV

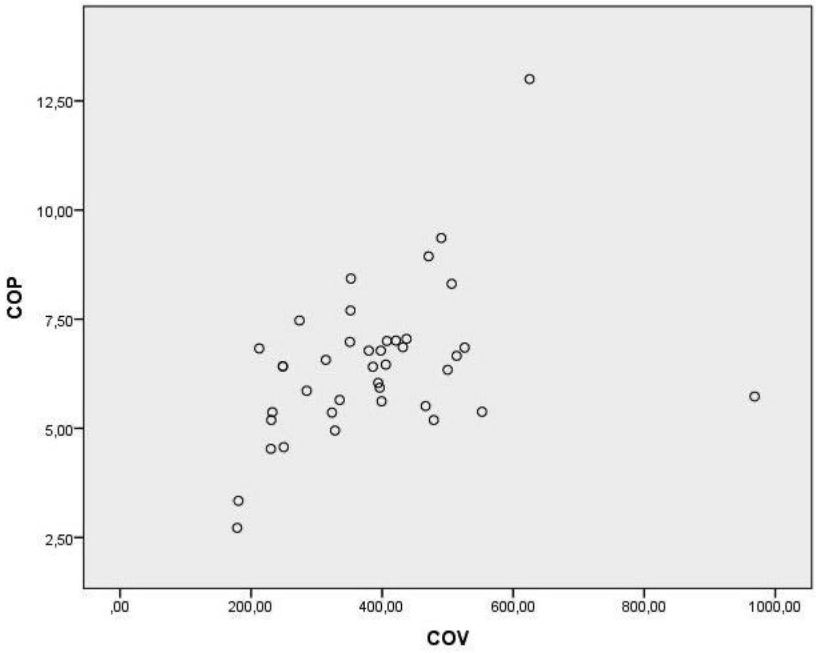

Non- parametric tests

Kolmogorov- Smirnov Test

		Body weigt	COV	COP
N		40	40	40
Parameters of normal distribution	Mean	2.951	387.0016	6.4392
	SD	.7527	144.35022	1.70082
Extreme difference	absolute	.197	.094	.185
	positive	.197	.094	.185
	negativ	-.149	-.075	-.106
Statistics for test		.197	.094	.185
Asymptotic significance (2 sides)		.000c	.200 c,d	.001c

a. Normal distribution
b. Calculated from data
c. Correction of significance after Lillefors
d. Lower limit of real significance

Kolmogorov Smirnov Adaptation Test

		Age	Body weight	Breed	COP	COV	slope	BCS
N		40	40	40	40	40	40	32
Normal Distribution [a,b]	Mean	12,63	2,951	2,78	6,4392	387,0016	,09496	5,5313
	SD	9,289	,7527	1,687	1,70082	144,35022	,023811	1,04679
Extreme Difference	Absolute	,327	,197	,427	,185	,094	,094	,194
	Positive	,327	,197	,427	,185	,094	,057	,194
	Negative	-,188	-,149	-,273	-,106	-,075	-,094	-,181
Statistic for test		,327	,197	,427	,185	,094	,094	,194
Asymptotic significance (2-sides)		,000[c]	,000[c]	,000[c]	,001[c]	,200[c,d]	,200[c,d]	,003[c]

a. Normal distribution

b. Calculated from data

c. Correction of significance after Lillefors

d. Lower limit of real significance

			COP	COV	Body weight	BCS	Total volume	Vol steps	L1	U1	L2	U2	Breed
Spearman-Rho	COP	Correlation coefficient	1,000	,436**	,169	,142	,212	-,140	,063	,026	,070	,097	-,063
		Sig. (2-sides)	.	,005	,298	,439	,190	,390	,702	,876	,667	,550	,697
		N	40	40	40	32	40	40	40	40	40	40	40
	COV	Correlation coefficient	,436**	1,000	,465**	,317	,886**	-,168	,319*	,185	,219	,347*	-,181
		Sig. (2-sides)	,005	.	,003	,077	,000	,300	,045	,253	,174	,028	,265
		N	40	40	40	32	40	40	40	40	40	40	40
	Gewicht	Correlation coefficient	,169	,465**	1,000	,501**	,586**	-,252	,274	,673**	,209	,731**	-,190
		Sig. (2-sides)	,298	,003	.	,003	,000	,117	,087	,000	,196	,000	,240
		N	40	40	40	32	40	40	40	40	40	40	40
	BCS	Correlation coefficient	,142	,317	,501**	1,000	,298	.	,182	,592**	-,005	,382*	,136
		Sig. (2-sides)	,439	,077	,003	.	,098	.	,318	,000	,979	,031	,459
		N	32	32	32	32	32	32	32	32	32	32	32
	Total volume	Correlation coefficient	,212	,886**	,586**	,298	1,000	-,281	,384*	,333*	,311	,477**	-,328*
		Sig. (2-sides)	,190	,000	,000	,098	.	,079	,014	,036	,050	,002	,039
		N	40	40	40	32	40	40	40	40	40	40	40
	Vol steps	Correlation coefficient	-,140	-,168	-,252	.	-,281	1,000	,040	,017	-,130	-,100	,302
		Sig. (2-sides)	,390	,300	,117	.	,079	.	,806	,916	,424	,538	,058
		N	40	40	40	32	40	40	40	40	40	40	40
	L1	Correlation coefficient	,063	,319*	,274	,182	,384*	,040	1,000	,432**	,002	,168	,077
		Sig. (2-sides)	,702	,045	,087	,318	,014	,806	.	,005	,992	,301	,638

		N	40	40	40	32	40	40	40	40	40	40	40
	U1	Correlation coefficient	,026	,185	,673**	,592**	,333*	,017	,432**	1,000	,065	,769**	,186
		Sig. (2-sides)	,876	,253	,000	,000	,036	,916	,005	.	,690	,000	,251
		N	40	40	40	32	40	40	40	40	40	40	40
	L2	Correlation coefficient	,070	,219	,209	-,005	,311	-,130	,002	,065	1,000	,136	-,155
		Sig. (2-sides)	,667	,174	,196	,979	,050	,424	,992	,690	.	,401	,338
		N	40	40	40	32	40	40	40	40	40	40	40
	U2	Correlation coefficient	,097	,347*	,731**	,382*	,477**	-,100	,168	,769**	,136	1,000	,058
		Sig. (2-sides)	,550	,028	,000	,031	,002	,538	,301	,000	,401	.	,724
		N	40	40	40	32	40	40	40	40	40	40	40
	Breed	Correlation coefficient	-,063	-,181	-,190	,136	-,328*	,302	,077	,186	-,155	,058	1,000
		Sig. (2-sides)	,697	,265	,240	,459	,039	,058	,638	,251	,338	,724	.
		N	40	40	40	32	40	40	40	40	40	40	40

**Correlation is significant (two sides) on the level of 0.01

*Correlation is significant (two sides) on the level of 0.05
